# Interactive Mixed Reality Simulation Enhances Student Knowledge and Ultrasound Interpretation in Sheep Pregnancy Diagnosis

**DOI:** 10.3390/vetsci13010080

**Published:** 2026-01-13

**Authors:** Madison Golledge, Katherine R. Seymour, Mike Seymour, Simon P. de Graaf

**Affiliations:** 1 Faculty of Science, The University of Sydney, Sydney, NSW 2006, Australiasimon.degraaf@sydney.edu.au (S.P.d.G.); 2 Business School, The University of Sydney, Darlington, NSW 2006, Australia

**Keywords:** ultrasound training, mixed reality, Apple Vision Pro, sheep pregnancy scanning, veterinary education, medical image education

## Abstract

Transitioning from theoretical learning to practical application remains a significant challenge for students in medical and veterinary science education, particularly in the context of medical imaging and ultrasound interpretation. Traditional lecture-based methods offer limited support for developing the spatial reasoning and technical skills required for ultrasound pregnancy diagnosis. This study evaluates the effectiveness of an interactive mixed reality (MR) training tool, Ewe Scan, delivered through the Apple Vision Pro, compared to traditional lecture-based instruction. Forty-two undergraduate students were randomly assigned to either a lecture-trained or MR-trained group and assessed immediately after training and again after six weeks. Results showed that MR-trained students significantly outperformed their lecture-trained peers in both immediate comprehension and retention over time, particularly in ultrasound interpretation skills. The MR-trained group also reported higher levels of engagement, confidence, and satisfaction with their training experience. These findings suggest that MR-based learning enhances educational outcomes by improving spatial understanding, increasing active engagement, and supporting knowledge retention. Integrating MR simulations into ultrasound education offers a scalable, ethical, and effective alternative to traditional training methods, contributing to advancements in medical imagery education.

## Simple Summary

Learning how to perform pregnancy ultrasound on sheep is an important skill for veterinary and animal science students, but it is difficult to teach using classroom lessons alone. Students must learn to turn a flat, two-dimensional ultrasound picture into an understanding of the 3D structures inside an animal, which many beginners find challenging. At the same time, opportunities to practice on live animals can be limited because of ethical and practical concerns. In this study, we tested the effectiveness of our training tool called Ewe Scan that uses a mixed reality headset to let students explore reproductive anatomy and practice pregnancy scanning in a realistic virtual environment. We compared students who used the Ewe Scan tool with those who received a traditional lecture. Students trained with mixed reality showed stronger understanding of ultrasound interpretation, remembered the material better six weeks later, and felt more confident and engaged during learning. These results show that mixed reality can improve training for important clinical skills in veterinary medicine while reducing pressure on live animals. As this approach supports learning of complex 3D ideas, it could also be adapted for other scientific or medical training where hands-on practice is difficult to provide.

## 1. Introduction

Medical and veterinary students alike face the challenge of transitioning from theoretical knowledge to hands-on clinical practice, especially in domains requiring the interpretation of complex medical imagery. Traditional teaching methods, particularly lecture-based instruction, rely heavily on 2D representations of anatomical structures such as textbooks, static images, and slides. The transition from 2D learning materials to working directly with patients, whether human or animal, presents a significant cognitive challenge. To address this gap, immersive technologies such as virtual reality (VR), augmented reality (AR), and mixed reality (MR) have emerged as powerful tools in health science education. These modalities offer interactive 3D visualizations that enhance spatial reasoning, reduce cognitive load, and improve procedural confidence during the transition to clinical practice [1,2,3,4,5]. Amongst the available technologies, the recently released Apple Vision Pro utilizes a very high resolution, low-latency head-mounted display (HMD) MR system that blends the immersive features of VR with the contextual layering of AR, allowing users to engage with both digital and physical environments simultaneously.

Ultrasound pregnancy diagnosis is a fundamental skill for veterinary and animal science students, playing a crucial role in reproductive management by enabling efficient flock management and early intervention when necessary [6]. However, mastering this technique presents significant challenges. It requires strong spatial reasoning, as students must interpret 2D ultrasound images in real-time to assess complex 3D internal structures [7]. Traditional lecture-based training methods, which rely primarily on verbal explanations and static diagrams, provide foundational knowledge but are inherently limited in preparing students for hands-on application [8]. Without prior practical experience, students often struggle with image interpretation and spatial reasoning, leading to frustration and inefficiency. These challenges are further exacerbated by ethical and logistical constraints that limit the availability of live animals for repeated hands-on practice. When students finally transition to real-world scanning, they must simultaneously manage probe positioning, diagnostic decision-making, and live animal handling, often in high-pressure environments where mistakes can compromise both learning and animal welfare. This combination of limited training opportunities, high cognitive load, and stress can negatively impact skill acquisition and confidence [1,8]. A structured, intermediate training tool that enhances spatial understanding before students engage with live animals could significantly improve educational outcomes.

Compared to traditional lecture-based training, MR-based learning offers several advantages. Reducing reliance on live animals for training aligns with modern principles of ethical education while still ensuring students develop the necessary technical skills [9,10]. MR also allows students to practice in a controlled, stress-free environment, free from the pressures of handling live animals during their initial learning stages. The use of interactive 3D visualizations improves spatial reasoning by helping students mentally translate 2D ultrasound images into 3D anatomical understanding, and the immersive nature of MR enhances student engagement and motivation [7,8,11]. The active learning nature of this modality supports not only better comprehension during training but also long-term recall of information [12]. The ability to practice independently and at one’s own pace further increases confidence, ensuring students are better prepared when transitioning to real-world application. Additionally, MR-based learning is scalable, allowing multiple students to train simultaneously, unlike live demonstrations or individualized training sessions, making ultrasound education more efficient and standardized.

This study evaluates the effectiveness of an Apple Vision Pro-based training program, Ewe Scan, designed to improve ultrasound pregnancy scanning skills in veterinary and animal science students. Specifically, it aims to compare MR-trained and traditionally lecture-trained students with the following metrics: (1) assessing scanning knowledge accuracy, (2) comparing short-term knowledge accuracy and long-term knowledge retention and (3) evaluating student engagement, confidence, and training perception. It is hypothesized that the interactive active learning achieved by the MR system will result in students performing better in all metrics than those in a lecture-based setting. By investigating the potential of MR to improve training in ultrasound diagnosis of pregnancy, this research contributes to the broader movement toward innovative, technology-driven solutions in veterinary education. If successful, MR-based training could serve as a scalable and ethical alternative or addition to traditional training methods, ultimately improving student preparedness and reducing reliance on live animal training in veterinary curricula.

## 2. Materials and Methods

### 2.1. Student Participants

A total of 51 first-year animal science undergraduate students enrolled in the Animal and Veterinary Bioscience (AVBS) course at the University of Sydney expressed interest in this study. Eligibility for this study was determined by a screening questionnaire using Research Electronic Data Capture (REDCap; Vanderbilt University, Nashville, TN, USA; version 14.3.13) hosted at the University of Sydney, a secure web-based application developed for data collection from participants for research studies [13]. Students were only accepted to this study if they had no prior ultrasound pregnancy diagnosis experience, no prior reproductive anatomy knowledge, and were willing to participate in both days of the study. Eligible and accepted students were randomly assigned to one of two groups: lecture-trained (control group) or MR-trained (experimental group).

Ethics approval was granted by The University of Sydney Human Research Ethics Committee (Protocol HE001177) on 30 September 2024, and all students provided informed consent prior to participation via REDCap. Students were excluded from the MR-trained group if they required glasses to read, as the Apple Vision Pro requires visually impaired users to use contact lenses or purchase custom lenses to use the device.

### 2.2. Training

**Figure 1:**
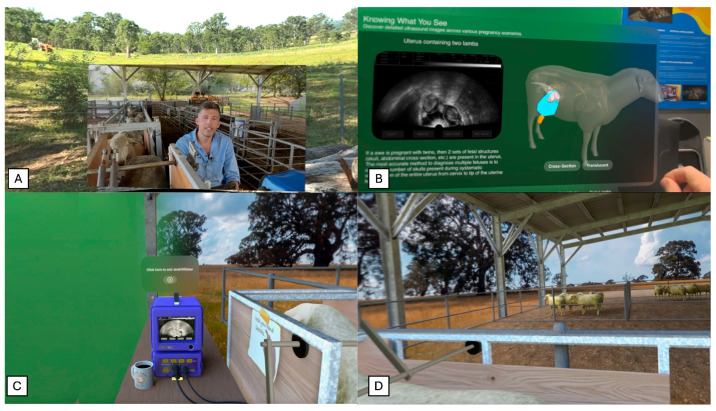
Training stages of mixed reality Ewe Scan Apple Vision Pro app, designed to teach sheep pregnancy scanning with ultrasound. (**A**) First stage: introductory video by University of Sydney Professor. (**B**) Second stage: 3D model of various pregnancy types (empty, single, twin) and information on important fetal structures. (**C**) Third stage: scanning simulator to practice probe positioning and fetal structure recognition. (**D**) Third stage: delivered in a mixed reality virtual sheep scanning environment.

Both training groups of students watched a pre-recorded introductory video, presented by a higher learning educator experienced in teaching reproductive anatomy and ultrasound diagnosis of pregnancy in sheep. The video covered topics including ultrasound equipment for sheep pregnancy scanning, ultrasound probe positioning on ewes, and key fetal structures that can be observed by ultrasound (Figure 1A). The video was filmed on a farm to show students what pregnancy scanning sheep would look like in a real-world setting.

#### 2.2.1. Lecture-Trained Group

Following watching the introductory video, the lecture-trained group (*n* = 22) received a slide presentation detailing sheep reproduction anatomy. The content of the presentation was designed to match the content the MR-trained group would be viewing when using Ewe Scan. Critically, the lecture-trained group was shown only currently available teaching materials (such as textbook diagrams) and not any of the interactive 3D models developed for the Apple Vision Pro. This content included 2D diagrams of maternal and fetal sheep anatomy. The pre-recorded introductory video and slides were all delivered by the same educator for 30 min in a classroom at The University of Sydney.

#### 2.2.2. MR-Trained Group

The MR-trained group (*n* = 19) received 30 min of individual training on the Ewe Scan app (developed by the University of Sydney research team, Sydney, NSW, Australia) on the Apple Vision Pro (Apple Inc., Cupertino, CA, USA). The students received a brief device orientation (3–5 min), explaining the hand commands as well as setting up personalized eye and hand tracking. This orientation was directed by a researcher with ultrasound scanning expertise and trained in Apple Vision Pro demonstration. The app guided the students through 3 stages of learning: an introductory video, an interactive 3D anatomical model explanation, and an interactive ultrasound simulation. As previously mentioned, the introductory video was the same as the one shown to the lecture-trained group. Although, the MR-trained group viewed the video from within the app in a spatial immersive, virtual paddock environment, shown in Figure 1A. The second stage displays a 3D model of a sheep which the students could interact with and move to view from any direction, shown in Figure 1B. The 3D model had three alternate versions, not pregnant (empty), pregnant with a single fetus, or pregnant with twin fetuses. Accompanying the 3D model were a number of key fetal structures that students could select and read about in more detail including the placentome, uterine wall, fetal skull, and amniotic sac. Selecting each structure displays the structure anatomically in 3D, 2D on ultrasound imagery, and a written description of visual traits of this structure. During the third stage of the app, shown in Figure 1C, the student is placed in a virtual environment where they can practice pregnancy scanning a virtual 3D sheep. This stage displays a typical sheep pregnancy scanning environment on farm, with a pregnancy scanning crate holding a sheep on the right side of the student and an ultrasound machine in front the student, displayed in Figure 1D. As students position the virtual probe on the sheep and begin to maneuver their hand, the ultrasound image updates dynamically based on their hand position. This allows students to practice probe positioning as well as understanding the spatial context of interpretating a 2D ultrasound image from the 3D anatomy of the sheep. A short demonstrative video of the Ewe Scan mixed reality app is provided as Supplementary Video S1.

### 2.3. Testing Procedures

All students were given the same test twice: once immediately after training (Immediate Testing) and again six weeks later (Follow-up Testing). Both tests administered to students are available as Supplementary File S1. Immediate Testing was completed in person on the University of Sydney campus directly after training and was used to determine comprehension between training groups. Follow-up Testing was conducted six weeks later and completed remotely via REDCap, allowing for analysis of knowledge retention over time. Students were given up to 30 min to complete the test. Tests were graded and cleaned manually due to the small sample size.

The educational content portion of the test was divided into two main sections: Reproductive Anatomy and Ultrasound Diagnosis, with five short-response questions in each section. These questions were designed to align with the specific learning outcomes targeted in each training method, directly reflecting the content covered in either the lecture-trained or MR-trained sessions. The Reproductive Anatomy section assessed understanding of anatomical structures of a pregnant ewe, while the Ultrasound Diagnosis section focused on interpretation of ultrasound images and fetal structure recognition.

**Figure 2:**
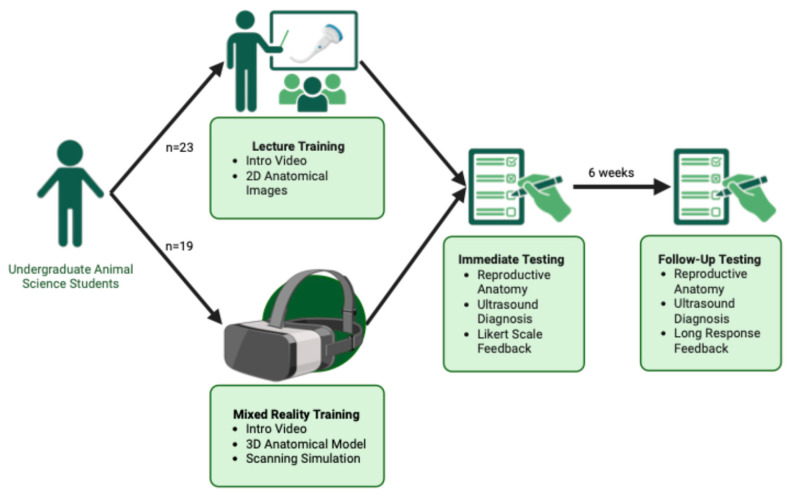
Study design and timeline for Mixed Reality vs. Lecture-based Training comparison. Students were randomly assigned to either MR training (*n* = 19) or lecture-based training (*n* = 22). Both groups viewed an introductory video, with MR-trained students completing additional 3D model exploration and a scanning simulation in the Apple Vision Pro. All students completed Immediate Testing after training and Follow-up Testing six weeks later, assessing anatomy knowledge, ultrasound interpretation, and training feedback. Created in BioRender. https://BioRender.com/ze0ze7n (Accessed on 31 July 2025).

Following these, a third section prompted students to reflect on their training experience. During Immediate Testing, students rated their confidence and engagement on a 1 to 5 scale. The Follow-up Testing phase included open-ended questions for students to provide feedback on their training, including the advantages and disadvantages of their assigned method and their preferred mode of instruction for future learning. Study design and timeline of testing is summarized in Figure 2.

### 2.4. Data Management and Cleaning

All test responses were collected via REDCap, which allows for de-identification of datasets using unique Record IDs. Personal identifiers (i.e., e-mail, names) were removed from the dataset prior to analysis. Data will be securely stored for 5 years and then destroyed.

Any students that did not complete both testing phases (Immediate and Follow-up) were removed from the dataset. Assessment scoring was performed by an independent rater blinded to group allocation. Objective scoring rubrics and consistent testing conditions were applied to minimize bias. Short answer content questions were recorded as either “Correct” or “Incorrect”, with incorrectly spelled answers recorded as a correct response. Scaled feedback questions were converted to a 1 to 5 scale for analysis. Short-response feedback responses were transformed into lists of theme code words for analysis.

### 2.5. Statistical Analysis

Data was cleaned and stored in Microsoft Excel, with all statistical analyses and visualization creation conducted in R (R Foundation for Statistical Computing, Vienna, Austria; version 4.4.1) using R-Studio (Posit Software, Boston, MA, USA; version 2024.09.0) [14]. Normality was assessed using histograms, Q-Q plots, and Shapiro–Wilk tests, revealing a right-skewed, non-normal distribution. Consequently, non-parametric tests were used. Summary statistics and boxplots were generated to describe data distribution.

The primary outcome variable was test score (%), calculated as both an overall test score and as section scores for Reproductive Anatomy and Ultrasound Diagnosis. Two key independent variables were used: Training Group (Lecture vs. MR) and Test Phase (Immediate vs. Follow-up). This design allowed for assessment of both between-group differences in learning outcomes and within-group changes over time, reflecting knowledge retention.

To evaluate differences in overall comprehension and retention between the two training methods, a Generalized Linear Mixed Model (GLMM) with a Gamma (log) distribution was applied to the total response accuracy data. Fixed effects included Training Group (lecture-trained vs. MR-trained), Test Phase (Immediate Testing vs. Follow-up Testing), and their interaction. A random intercept for Student ID was included to account for repeated measures. Post hoc comparisons using estimated marginal means (emmeans) were conducted to further interpret pairwise group differences, identifying whether MR-based training led to significantly greater learning gains and retention over time compared to traditional lecture-based instruction. Comparisons were performed on the log-transformed scale to match model assumptions, and results were back-transformed for interpretation and reporting in figures.

To determine the effect of test section on test score, a Kruskal–Wallis test was employed, with a test statistic of H quantifying the difference in rank distributions. Wilcoxon Rank-Sum tests were used to compare training groups for each testing phase. Median scores, mean scores, and distributions were visualized in a boxplot.

To assess student-rated engagement and confidence, scaled feedback scores on the Likert scale were compared between groups using Wilcoxon Rank-Sum tests for each question. Bonferroni-adjusted *p*-values were applied to account for multiple comparisons. Mean scores and distributions were visualized using a bar chart.

Short-response student feedback data was analyzed using mixed methods. Descriptive statistics were used to summarize the frequency of commonly reported advantages and disadvantages of each training method. The number of positive (pros) and negative (cons) descriptors provided per student was compared between groups using Wilcoxon rank-sum tests. The total number of pros and cons reported across groups was analyzed using Pearson’s Chi-squared test. Training method preferences were evaluated using a binomial test to determine whether Vision Pro was selected significantly more often than other options. Results were visualized through bar charts and frequency distributions.

## 3. Results

### 3.1. Study Implementation

A total of 51 students expressed interest in participating in the study. Of these, 44 students completed the initial training session and Test 1 (lecture-trained: *n* = 23; MR-trained: *n* = 21). 41 students completed both Immediate and Follow-up Testing and were included in the final analysis, resulting in final group sizes of *n* = 22 (lecture-trained) and *n* = 19 (MR-trained). No major technical issues were encountered during MR training, and no participants reported motion sickness. All students were able to use the device effectively for the short-term training session, despite the device being shared across multiple users. Although students were allocated up to 30 min to complete each test, most completed the questionnaire in under 20 min.

### 3.2. Overall Performance Across Training Group

#### 3.2.1. Comprehension

Descriptive statistics in Table 1 show that in raw percentage terms, the MR-trained group reported higher test scores during both testing phases, with a higher median, higher mean, and lower variability compared to the lecture-trained group.

To assess whether these differences were statistically meaningful after accounting for repeated measures, a GLMM was applied. In Table 2, results from this analysis confirmed that MR-trained students scored significantly higher than their lecture-trained peers across both testing phases (*p* = 0.0155).

**Figure 3:**
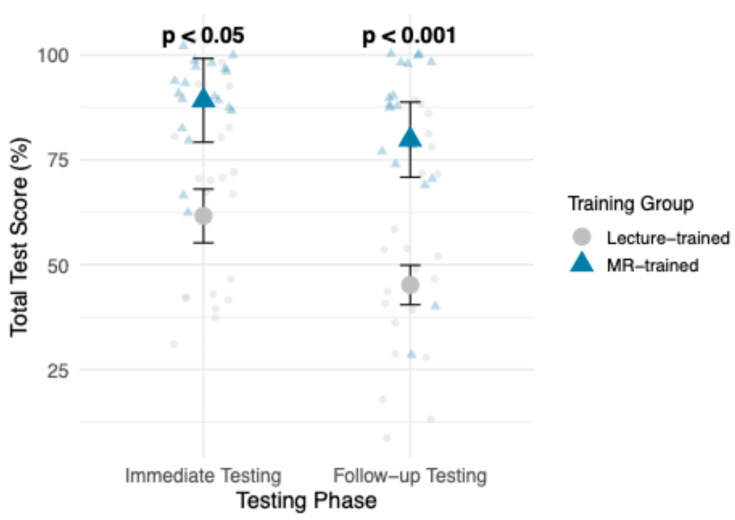
Estimated marginal means (emmeans) of overall test scores (%) for lecture-trained vs. MR-trained, across both Testing Phases (Immediate Testing and Follow-up Testing). *p*-value ranges reflect pairwise contrast results between training groups.

The emmeans analysis further supported this finding, demonstrating that the MR-trained group had higher estimated marginal means during both testing phases, shown in Figure 3. The pairwise contrast analysis between training groups revealed a significant difference between groups at both time points. During Immediate Testing, MR-trained students significantly outperformed lecture-trained students (82.9% vs. 61.6%, *p* = 0.0155). This trend continued in Follow-Up Testing, with increased significance (79.8% vs. 45.2%, *p* = 0.0002).

#### 3.2.2. Knowledge Retention

In comparing the change in raw mean test score in Table 1 between Immediate Testing and Follow-up Testing for each group, MR-trained group had a smaller decline in mean overall test score of 7.37%, compared to the larger 14.09% decline in the lecture-trained group. In Table 2, GLMM results confirmed that overall test scores in both training groups declined over time, with Follow-up Testing scores significantly lower than Immediate Testing (*p* < 0.001). However, a significant Training Group × Testing Phase interaction (*p* = 0.0414) suggests that the decline in scores was less pronounced for MR-trained students.

#### 3.2.3. Performance by Test Section

**Figure 4:**
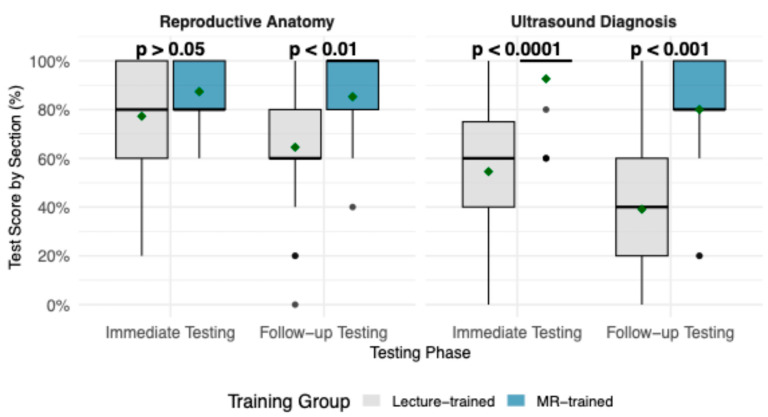
Boxplots demonstrating test score (%) by section, Reproductive Anatomy and Ultrasound Diagnosis, compared between lecture-trained and MR-trained groups, across testing phases. Boxes represent the interquartile range, with the horizontal line indicting the median; whiskers extend to the minimum and maximum values, and individual points represent outliers. Mean annotated with a green diamond point. *p*-values from Wilcoxon rank-sum tests comparing training groups are shown for each testing phase.

Performance trends across Reproductive Anatomy and Ultrasound Diagnosis sections are shown in Figure 4. MR-trained students reported higher mean and median scores across both sections and test phases. In Reproductive Anatomy, group mean scores were comparable during Immediate Testing (87.4% MR vs. 77.3% lecture, *p* > 0.05), but a clearer gap emerged at Follow-up Testing (85.2% vs. 64.5%, *p* < 0.01), indicating stronger retention in the MR-trained group. In Ultrasound Diagnosis, the differences were more pronounced as MR-trained students outperformed lecture-trained students at both Immediate Testing (92.6% vs. 54.5%, *p* < 0.0001) and Follow-up Testing (80.0% vs. 39.1%, *p* < 0.001). A Kruskal–Wallis Test confirmed that the effect of training group on test scores was strongest in the Ultrasound Diagnosis section (H = 30.76, *p* < 0.0001), followed by Reproductive Anatomy (H = 7.30, *p* < 0.01).

### 3.3. Student Training Experience

**Figure 5:**
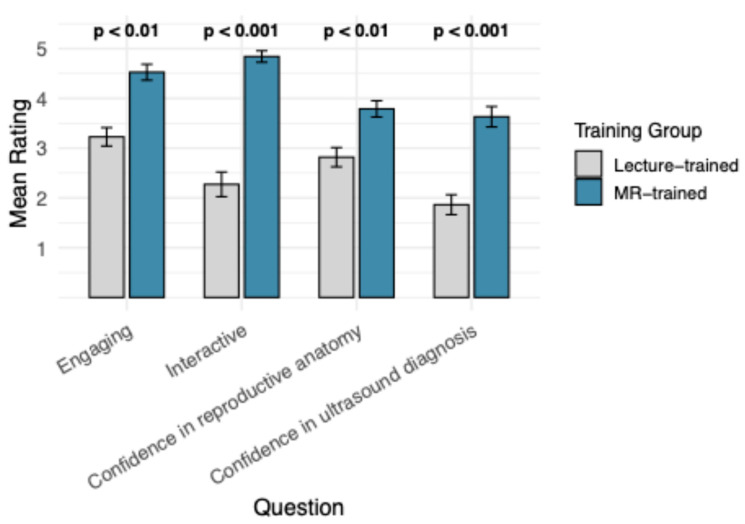
Mean student score of Likert feedback questions regarding their training experience, compared between training groups. Wilcoxon Rank Sum Test *p*-value ranges annotate significant differences between training groups for each feedback question. Bars demonstrate Standard Error of the Mean.

Student feedback ratings for training engagement and confidence differed significantly between groups are demonstrated in Figure 5. The MR-trained group reported significantly higher ratings across all assessed categories compared to the lecture-trained group. Students in the MR-trained group rated their training session as significantly more interactive (*p* < 0.001) and engaging (*p* < 0.01), compared to the lecture-trained students. Similarly, confidence levels in ultrasound diagnosis (*p* < 0.001) and reproductive anatomy (*p* < 0.01) were significantly higher in the MR-trained group, compared to lecture-trained students.

**Figure 6:**
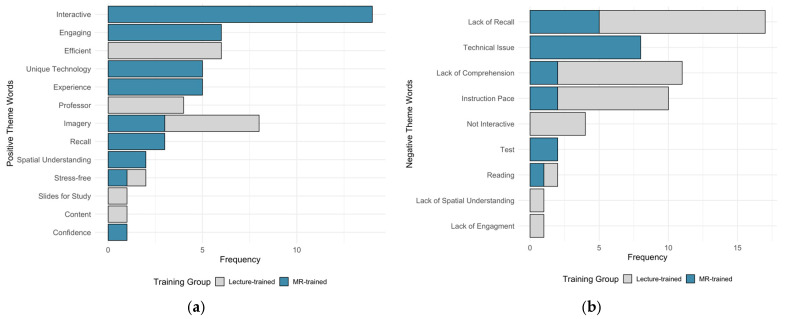
Student long response feedback regarding (**a**) positive and (**b**) negative aspects of their assigned training method. Comparing number of instances of each theme code word in student response per group.

Student responses regarding the most common advantages and disadvantages of each training method are summarized in Figure 6. The most frequently mentioned benefits in the MR-trained group included interactive, engaging, and experience, while the lecture-trained group most commonly noted “efficient” and “imagery”, shown in Figure 6a. The most frequent disadvantages reported in the MR-trained group was “technical issues”, whereas the lecture-trained group frequently mentioned “lack of comprehension” and “instruction pace”, shown in Figure 6b. Both training groups reported a difficulty with “lack of recall” Figure 6b.

**Figure 7:**
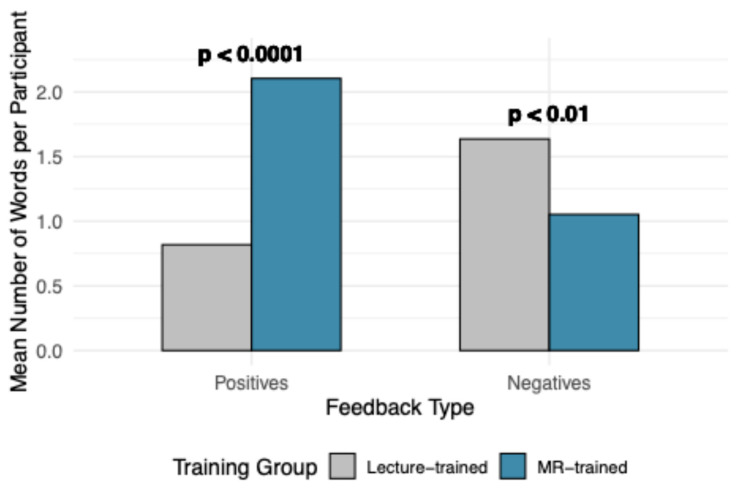
Summaries of theme code words by feedback type and number of theme code words. Represented by mean number of mentions of positive and negative words, with *p*-value indicating significance between training groups, determined by Wilcoxon Rank Sum Test.

The number of positive and negative descriptors used per student was analyzed and summarized in Figure 7. The MR-trained group reported significantly more positive descriptors than the lecture-trained group (*p* < 0.0001). Conversely, the lecture-trained group used significantly more negative descriptors compared to the MR-trained group (*p* < 0.01). The total number of descriptors used also differed between groups, with the MR-trained group providing more positive words and the lecture-trained group providing more negative words (*p* < 0.001).

**Figure 8:**
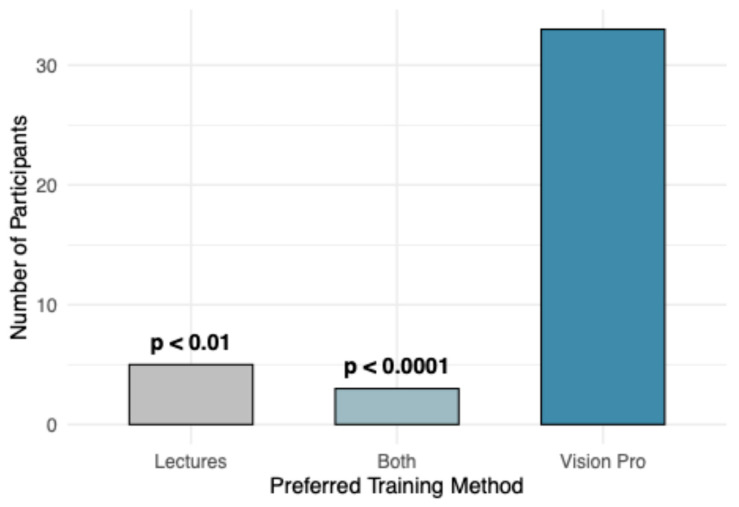
Comparison of number of students that gave responses when asked “If you could choose between the training methods (lecture or Vision Pro app), which would you prefer for learning similar content in the future?” *p*-value ranges represent results of binomial test, comparing number of students that chose Vision Pro method, to Lectures and Both.

Student preferences for future training methods are presented in Figure 8. When asked, “If you could choose between the training methods (lecture or Vision Pro app), which would you prefer for learning similar content in the future?”, the majority of students (80.5%) indicated a preference for the MR-trained method. Although only the experimental group experienced the MR-trained firsthand, lecture-trained students were provided with a brief description of the app’s functionality before being asked about their future preferences. Binomial tests revealed that “Vision Pro” was selected significantly more often than “Both” (*p* < 0.0001) and “Lectures” (*p* < 0.01). When stratified by training group, both MR-trained and lecture-trained students expressed a stronger preference for Vision Pro. A Chi-square test indicated no significant effect of training group on training preference (*p* > 0.05).

## 4. Discussion

Our results suggest that MR-trained students comprehended sheep pregnancy scanning topics and retained this knowledge significantly better than traditional lecture-trained students, confirming our hypothesis. MR-trained students showed a significant dominance in both testing phases, reporting 24.09% higher results in immediate testing and growing to a 30.81% disparity in follow-up testing 6 weeks later. This demonstrates that the Ewe Scan app was successful in improving not only their understanding of the concepts immediately after training but also their retention of concepts, aligning with previous literature [8,15,16]. The interactive 3D model of a pregnant sheep allowed students to manipulate structures, compare pregnancy types, and actively engage with the content, which likely contributed to superior retention and comprehension.

Furthermore, the immersive scanning experience provided real-world context and improved students’ spatial understanding of ultrasound scanning. The ability to dynamically adjust the ultrasound image in response to probe movement likely aided in hand-eye coordination and transducer control, a skill gap often noted in ultrasound education [7]. This aligns with prior findings that interactive, spatially immersive learning environments improve procedural skill development [2]. The significant improvement in scores among MR-trained students supports the well-established positive relationship between active learning and educational outcomes [12]. Passive learning methods, such as traditional lectures, often lead to greater cognitive load and lower knowledge retention over time [8], which is reflected in the significant decline in overall test scores observed in lecture-trained students.

MR-trained students reported significantly higher levels of engagement and interactivity during their training. These students reported more advantages for their training method, noting the unique technology and detailed visualizations and enjoying their engaging experience. This positive perception of the technology mirrors the student reaction to VR and AR training in similar experimental trials [17,18,19,20]. Conversely, lecture-trained students reported lower engagement and greater difficulty keeping up with the content. This aligns with previous research demonstrating that passive learning leads to disengagement and reduced retention [12]. However, some students valued the efficiency and structured pace of lecture-based training, suggesting that a hybrid model combining MR and lectures may provide an optimal learning experience.

The most significant improvements were observed in ultrasound diagnosis, where MR-trained students consistently outperformed the lecture-trained group. This was expected, as ultrasound scanning requires strong spatial skills and the ability to translate 3D anatomical structures into 2D ultrasound images. The Ewe Scan app provided a unique opportunity to view a 3D sheep model alongside corresponding ultrasound slices, directly addressing the major challenge of ultrasound interpretation [8]. This method aligns with Hart, Wood and Weng [9], who found that hands-on, interactive anatomy learning significantly enhances both conceptual understanding and student confidence. By engaging in self-directed exploration, students in the MR-trained group could reinforce their knowledge through visual, spatial, and interactive cues, rather than relying on passive observation of diagrams. Lecture-trained students, in contrast, relied on 2D images and verbal descriptions, making it difficult to develop spatial intuition. This finding is supported by a study that demonstrated that VR training significantly improved visuospatial skills in surgical trainees compared to traditional lectures [16].

While MR-trained and lecture-trained students performed similarly in immediate testing, MR-trained students had better content recall in the follow-up test, reporting a decline in accuracy of only 2.11%, compared to the 12.72% decline in lecture-trained students. The ability to manipulate a 3D anatomical model in the Ewe Scan app may have helped students understand not only the spatial relationships between reproductive structures but also how those structures change across different pregnancy stages. Unlike traditional static 2D diagrams, which require students to mentally reconstruct these spatial relationships, VR-based anatomy training provides real-time, interactive exploration [3]. This aligns with findings from a study that demonstrated that VR simulation in anatomy training enhanced student comprehension by allowing them to manipulate and explore complex biological structures dynamically [17].

The effectiveness of MR-based training in ultrasound diagnosis and reproductive anatomy was demonstrated through both improved assessment outcomes and significantly higher self-reported confidence among students. Students who underwent MR training expressed greater confidence in their ability to identify reproductive anatomy and recognize fetal structures on ultrasound. These findings support the conclusion that MR training facilitates the integration of theoretical knowledge with practical skills, thereby better preparing students for the transition to live animal practice. Our findings align with and expand on prior simulation-based ultrasound training research [15,21,22]. In contrast to these studies, our MR app trained completely naive students without lecture input, included long-term retention, and emphasized educational outcomes such as engagement and confidence in addition to accuracy.

Student perceptions of each training modality further contextualize these findings and are reflected in the themed response patterns. MR-trained students most frequently associated their experience with themes such as engaging, interactive, unique technology, and spatial understanding, highlighting the perceived value of immersive, hands-on learning and dynamic linkage between probe positioning and ultrasound imagery. In contrast, lecture-trained students more commonly identified positive aspects related to imagery, efficiency, and instructor-led explanation, reflecting the familiarity and scalability of traditional teaching approaches. Analysis of negative responses revealed that lecture-trained students more frequently reported challenges related to lack of recall, lack of comprehension, and lack of spatial understanding, whereas MR-trained students reported fewer negative themes overall, with occasional references to small issues due to the novelty of the tech. Together, these patterns suggest that MR-based training was perceived as more engaging and supportive of spatial learning, while lecture-based training retained advantages in efficiency and instructional structure, reinforcing the value of MR as a complementary educational tool rather than a replacement for traditional teaching.

One of the major advantages of MR-based training is its potential to reduce reliance on live animals in veterinary education. Alternatives such as physical models and simulations have long been explored as ethical replacements for live animal training [9]; however, traditional models lack interactivity and scalability. MR provides a modern, scalable alternative that aligns with the 4Rs principle (Replacement, Reduction, Refinement, Responsibility) in animal research and education. Improving student preparation prior to live animal use can lead to better animal welfare outcomes, particularly in high-pressure training environments where student inexperience may cause unnecessary stress to animals. MR can ensure that students reach a basic competency level before working with live patients, reducing handling errors and minimizing stress for both students and animals. A previous study found that VR-trained students performed ultrasound scans more efficiently, with higher-quality imagery and increased confidence compared to traditional training [15]. While this study did not directly assess real-world scanning performance, it is expected that MR-trained students would demonstrate similar efficiency improvements, leading to shorter scan times per animal and reduced stress to both animals and students during pregnancy scanning training.

While MR training offers significant advantages, some practical considerations remain. The cost of MR headsets, particularly high-end devices such as the Apple Vision Pro, may limit widespread adoption. Further, standardization of MR-based curricula remains a challenge due to its novelty [1].

This study was conducted at a single institution using a relatively homogenous cohort of first-year undergraduate students. While this approach helped isolate the effects of the MR intervention, it also limits generalizability. Individual differences in student motivation or interest were minimized through random assignment and standardized baseline instruction, although they cannot be fully excluded. Future research could evaluate MR training across diverse academic levels, institutions, and cultural contexts. In addition, while this study assessed knowledge, confidence, and ultrasound interpretation accuracy, further work is needed to evaluate real-world scanning proficiency, such as the ability to locate fetal structures in live animals and complete diagnostic tasks under time constraints.

Development of Ewe Scan will continue, with improvements focused on refining the immersive scanning experience. Enhancements such as adding a virtual transducer in the student’s hand, requiring correct probe placement before scanning, and improving motion tracking could further enhance realism. Additionally, gamification features, such as fetal number estimation scoring, could increase student engagement and provide a more interactive assessment tool. Beyond ultrasound training, MR-based learning has broader applications in veterinary education, including disease diagnosis and surgical procedures. Integrating MR into a hybrid learning model alongside traditional lectures may provide the most balanced approach, maximizing both theoretical knowledge and hands-on experience.

## 5. Conclusions

MR-trained students significantly outperformed traditionally trained students in most educational and perception metrics, demonstrating higher test scores in both reproductive anatomy and ultrasound interpretation, greater engagement, and increased confidence in ultrasound scanning. These findings reinforce the importance of active learning, spatial interactivity, and immersive visualization in medical imagery education. Given the success of Ewe Scan, we recommend that AR/VR training be incorporated as a core component in ultrasound education, ideally in a hybrid model alongside traditional lectures to balance structured instruction with hands-on, immersive learning. Future MR applications could extend beyond ultrasound pregnancy scanning to the interpretation of other imaging modalities (Magnetic Resonance Imaging, Computer Tomography), surgery practice, and procedural skills training, advancing medical and veterinary education into the modern era of the 4Rs. Investing in interactive, technology-driven training modalities will not only enhance student learning outcomes, engagement, and confidence but also promote more ethical, effective approaches to medical imagery education.

## Figures and Tables

**Table 1:** Summary statistics of overall test score by training group and testing phase, reporting number of observations (*n*), mean overall test score, median overall test score, and standard deviation (SD).

Training Group	Testing Phase	*n*	Median Test Score	SD
Lecture-trained	Immediate Testing	22	65.91%	21.18%
	Follow-Up Testing	22	51.82%	24.62%
MR-trained	Immediate Testing	19	90.00%	11.06%
	Follow-Up Testing	19	82.63%	19.96%

**Table 2:** Generalized linear mixed model (Gamma distribution with log link) assessing the effects of training group (lecture-trained vs. MR-trained), testing phase (Immediate vs. 6-week Follow-up), and their interaction on test scores. Lecture-trained students at Immediate Testing were used as the reference category for all contrasts.

	Estimate (log)	t-Value	*p*-Value
Training group (MR vs. Lecture)	0.36994	2.421	*p* = 0.0155
Testing phase (Follow-up vs. Immediate)	−0.31072	−4.614	*p* < 0.001
Training group X Testing phase	0.19973	2.039	*p* = 0.0414

## Data Availability

The data presented in this study are available on request from the corresponding author due to ethical restrictions. The dataset contains individual test responses from undergraduate students, and consent was obtained only for the publication of summary, de-identified results rather than public release of raw data.

## Supplementary Materials

The following supporting information can be downloaded at: https://www.mdpi.com/article/10.3390/vetsci13010080/s1, File S1: Immediate and Follow-up Tests; Video S1: Demonstration of the Ewe Scan mixed-reality ultrasound training application.

## Abbreviations

The following abbreviations are used in this manuscript:

MR	Mixed Reality
VR	Virtual Reality
AR	Augmented Reality
HMD	Head-Mounted Display
AVBS	Animal and Veterinary Bioscience
REDCap	Research Electronic Data Capture
GLMM	Generalized Linear Mixed Model
R	Refers to the R programming language
