# Supplementary material for: Interactive Mixed Reality Simulation Enhances Student Knowledge and Ultrasound Interpretation in Sheep Pregnancy Diagnosis
Source: Vet Sci. 2026 Jan 13;13(1):80. doi: 10.3390/vetsci13010080 (PMC12846684; doi:10.3390/vetsci13010080)
Supplement: Supplementary file 1 [file vetsci-13-00080-s001.zip › FileS1_Follow-up and Immediate Tests.pdf]

Immediate Test

Reproductive Anatomy

Scanners must have a knowledge of reproductive anatomy to understand what they are observing in the ultrasound imagery. Identify which structures are being highlighted in this model of a pregnant ewe.

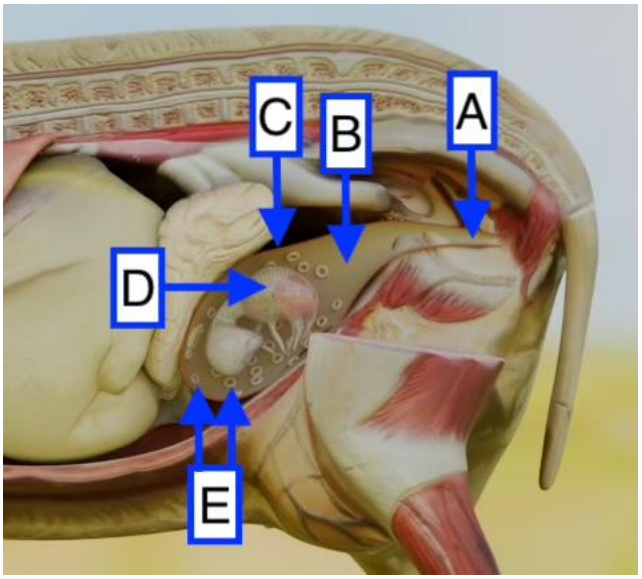

1. Write which structure 'A' is pointing to.
2. Write which structure 'B' is pointing to.
3. Write which structure 'C' is pointing to.
4. Write which structure 'D' is pointing to.
5. Write which structure 'E' is pointing to.

Fetal Structures via Ultrasound

Identifying fetal structures is the cornerstone of accurate pregnancy diagnosis by ultrasound. Identify which structures are being highlighted in each image.

6. Write which fetal structure the arrow is pointing to in this ultrasound picture.

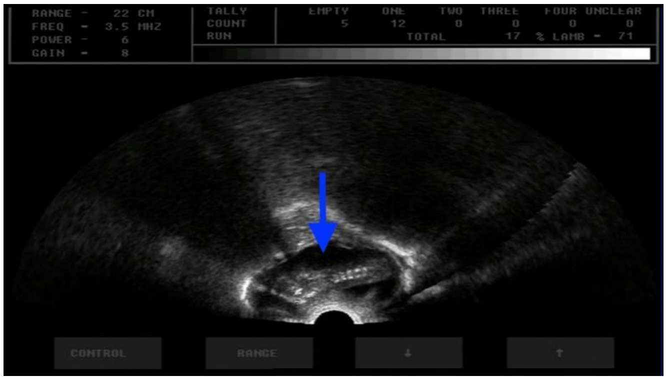

7. Write which fetal structure is circled in this ultrasound picture.

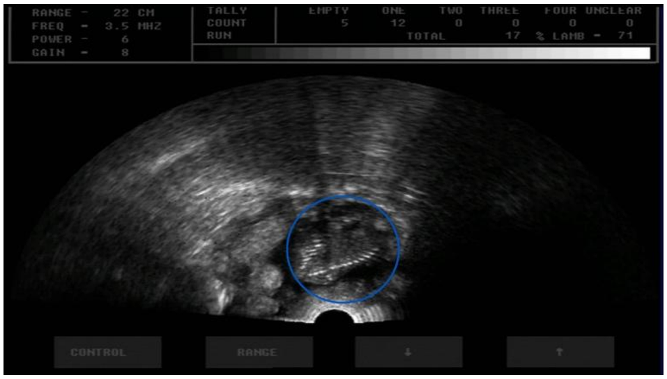

8. Write which fetal structure is circled in this ultrasound picture.

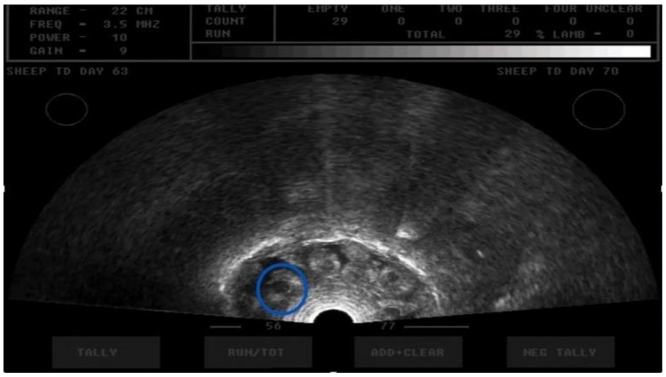

9. Write which fetal structure is circled in this ultrasound picture.

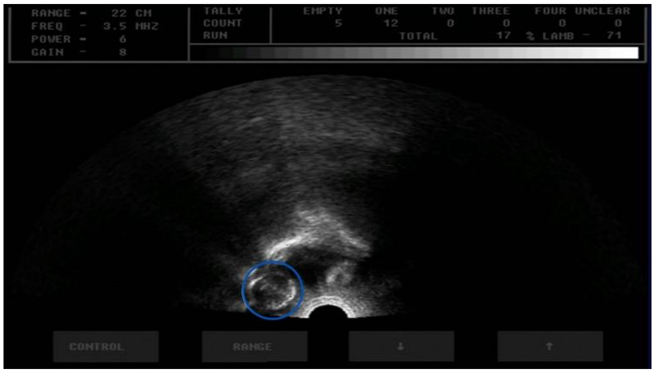

10. Write which fetal structure is the arrow is pointing to in this ultrasound picture.

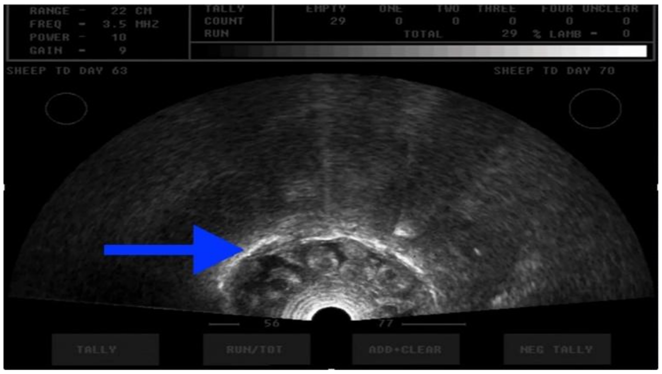

Training Experience

Your training experience, as well as your level of confidence regarding the content, is very important for us to understand the degree success of training. Please let us know how you found your training experience. Please be brutally honest, we won't be offended.

	Strongly Agree	Agree	Neutral	Disagree	Strongly Disagree
My training session was very engaging.					
My training session was very interactive.					
I feel confident in my ability to place a probe on a live sheep after training.					
I feel confident in my ability to recognize fetal structures on ultrasound imagery after training.					
I feel confident in my understanding of female reproductive anatomy of sheep after training.					

Follow-Up Test

Reproductive Anatomy

Scanners must have a knowledge of reproductive anatomy to understand what they are observing in the ultrasound imagery. Identify which structures are being highlighted in this model of a pregnant ewe.

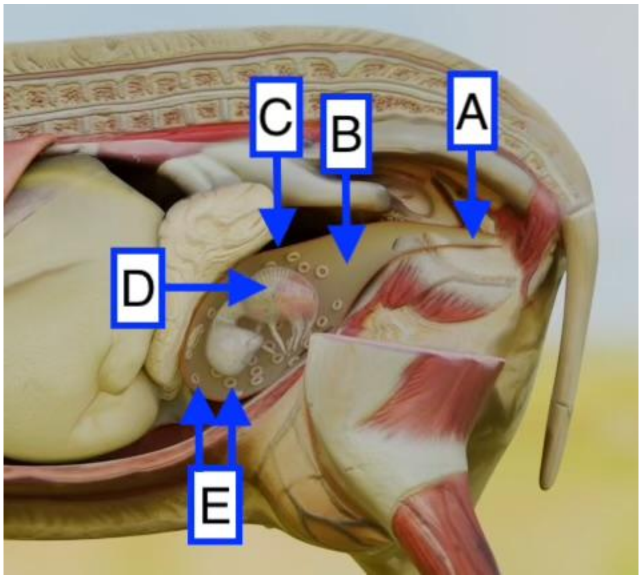

1. Write which structure 'A' is pointing to.
2. Write which structure 'B' is pointing to.
3. Write which structure 'C' is pointing to.
4. Write which structure 'D' is pointing to.
5. Write which structure 'E' is pointing to.

Fetal Structures via Ultrasound

Identifying fetal structures is the cornerstone of accurate pregnancy diagnosis by ultrasound. Identify which structures are being highlighted in each image.

6. Write which fetal structure is circled in this ultrasound picture.

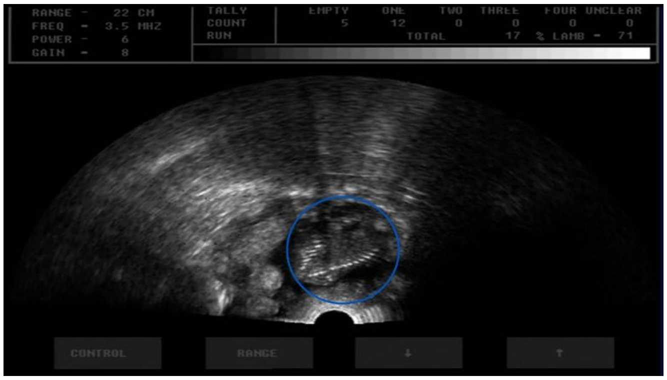

7. Write which fetal structure the arrow is pointing to in this ultrasound picture.

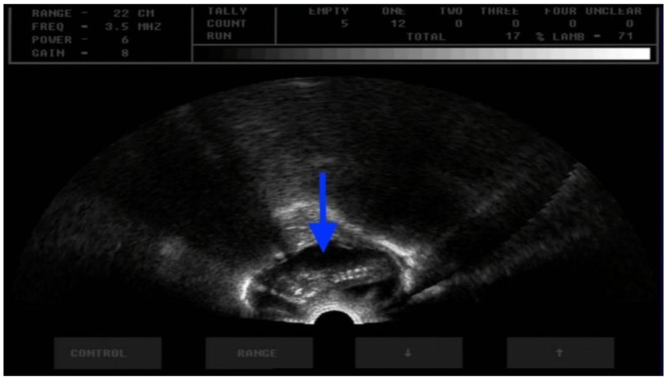

8. Write which fetal structure is circled in this ultrasound picture.

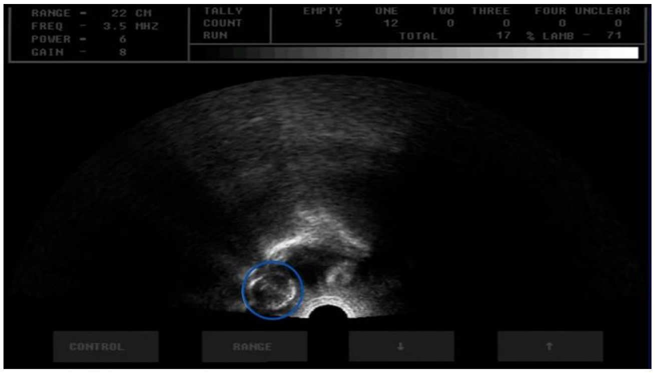

9. Write which fetal structure is the arrow is pointing to in this ultrasound picture.

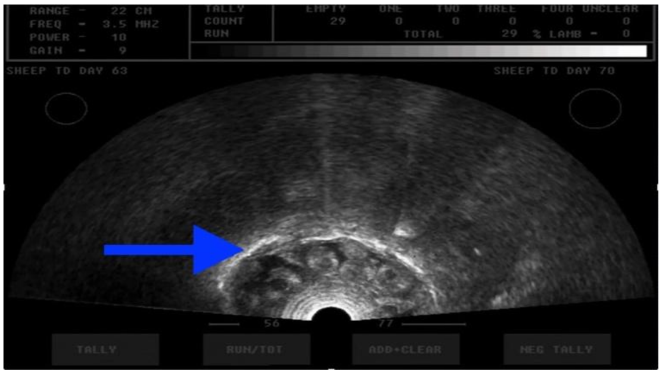

10. Write which fetal structure is circled in this ultrasound picture.

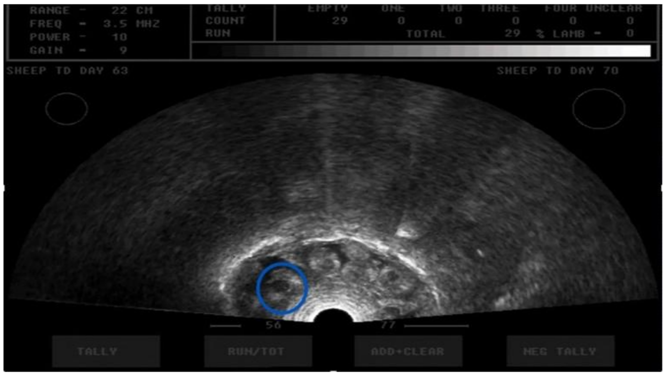

Training Experience

Please let us know how you found your training experience. This is your chance to give us feedback on the training methods and influence how you learn in the future. These responses will not be linked to your name, just like all your test results. If you were in the Vision Pro group, no need to report about the bugginess about stage 3, as we are aware of this issue and will fix this prior to releasing the app.

11. What did you like most about the training method you were allocated?
12. What did you find challenging or difficult about the training method?
13. Is there anything about the training that you feel could be improved? If so, what and how?
14. Were there any specific concepts or skills you feel were not adequately covered?
15. If you could choose between the training methods (lecture or Vision Pro app), which would you prefer for learning similar content in the future, and why?
